# Case Report: Preserved umbilical cords underscore family histories of inborn errors of immunity

**DOI:** 10.3389/fimmu.2025.1605857

**Published:** 2025-07-08

**Authors:** Madoka Nishimura, Dan Tomomasa, Rika Suzuki, Futaba Miyaoka, Hirokazu Kanegane

**Affiliations:** ^1^ Department of Pediatrics and Developmental Biology, Graduate School of Medical and Dental Sciences, Institute of Science Tokyo, Tokyo, Japan; ^2^ Department of Pediatrics, Graduate School of Medical Sciences, Kumamoto University, Kumamoto, Japan; ^3^ Department of Pediatrics, Nara Prefecture General Medical Center, Nara, Japan; ^4^ Department of Pediatrics, Kawaguchi Municipal Medical Center, Saitama, Japan; ^5^ Department of Child Health and Development, Graduate School of Medical and Dental Sciences, Institute of Science Tokyo, Tokyo, Japan

**Keywords:** inborn errors of immunity, umbilical cord, X-linked agammaglobulinemia, *Bruton tyrosine kinase*, family history

## Abstract

A history of susceptibility to infections and a family history of death because of unexplained infections during infancy are helpful in diagnosing inborn errors of immunity (IEIs). However, infections can occur because of various reasons, and determining whether the underlying disease is undoubtedly an IEI is implausible at present. In Japan, preservation of the umbilical cord at birth is customary. Two patients were suspected of having X-linked agammaglobulinemia (XLA); the patients were ultimately diagnosed with XLA based on the history of susceptibility to infections and family histories of deaths of maternal uncles because of infections during infancy. DNA was extracted from umbilical cords that had been preserved for approximately 50 years. The affected children harbored the same *Bruton tyrosine kinase* (*BTK*) variants as those detected using the umbilical cord samples of their maternal uncles. Analysis of preserved umbilical cords can help in ascertaining a family history of IEIs.

## 1 Introduction

Inborn errors of immunity (IEIs) are rare genetic disorders caused by monogenic defects in immunocompetent cells. More than 500 IEIs are known (1). Although IEIs are sometimes associated with malignancies, autoimmune diseases, autoinflammatory diseases, and allergies, many patients are susceptible to various infections. IEI is a genetic disease, and the diagnosis relies not only on the patient’s own history of susceptibility to infections but also on a family history of fatal infections during childhood. Of the 10 warning signs of suspected IEIs, family history has been reported to be the most useful for diagnosis of the disease (2). Infectious diseases can be caused by a variety of agents and, in rare cases, even in healthy individuals, they can be fatal. In Japan and few other countries, preservation of the umbilical cords is customary at birth (3). DNA extracted from preserved umbilical cords can be used to diagnose infectious diseases (3–8) and genetic disorders (9–13). Here, we describe two families with X-linked agammaglobulinemia (XLA). DNA was extracted from the umbilical cords of maternal uncles who died of infectious diseases during infancy approximately 50 years ago. The affected children harbored the same *Bruton tyrosine kinase (BTK)* variant as those detected using the umbilical cord samples of their maternal uncles.

## 2 Materials and methods

### 2.1 Flow cytometric analysis of BTK expression in monocytes

Monocytes were stained to detect intracellular BTK expression according to a previously described method (14). Peripheral blood mononuclear cells (PBMCs) were separated by Lymphoprep gradient centrifugation (Axis Shield Diagnostics Ltd., Dundee, Scotland). PBMCs were labeled with phycoerythrin-conjugated anti-CD14 (IgG2b; Becton Dickinson, Franklin Lakes, NJ, USA) monoclonal antibodies (mAbs). The cells were fixed in 4% paraformaldehyde in phosphate-buffered saline for 15 min and permeabilized with 0.1% Triton X-100 for 5 min. Subsequently, the cells were incubated with anti-BTK (clone 10E10; OriGene, Rockville, MD, USA) or isotype mAbs and subsequently reacted with fluorescein isothiocyanate (FITC)-conjugated anti-mouse IgG2a (Southern-Biotech, Birmingham, AL, USA). The stained cells were using a BD LSRFortessa flow cytometer (Becton Dickinson). Data were analyzed using FlowJo flow cytometry analysis software (FlowJo LLC, Ashland, OR, USA).

### 2.2 DNA extraction from whole blood and preserved umbilical cord

Portions of the umbilical cords, which were preserved for approximately 50 years (**Supplementary Figure 1**), were crushed using a rotor. Animal tissue lysis (ATL) buffer and protein kinase were added, and the samples were incubated overnight at 56 °C. DNA was extracted from whole blood and umbilical cords using the QIAamp DNA Blood Mini Kit (QIAGEN, Hilden, Germany).

### 2.3 Genetic analysis

Targeted gene panel sequencing of analysis of B-cell deficiencies (*BTK, IGHM, IGLL1, CD79A, BLNK, PIK3CD, PIK3R1, TCF3, SLC39A7, TRNT1, IKZF1, IKZF3*) were performed at the Kazusa DNA Research Institute (Kisarazu, Chiba, Japan). Genomic DNA extracted from whole blood samples of probands was enriched for protein-coding exons and adjacent intron-exon boundaries of target genes using either hybridization-based capture. Subsequent sequencing was performed by short fragment sequencing using an Illumina next generation sequencing system (Illumina, San Diego, CA, USA). The *BTK* variants were verified using Sanger sequencing.

## 3 Results

### 3.1 Case presentation

**Figure 1:**
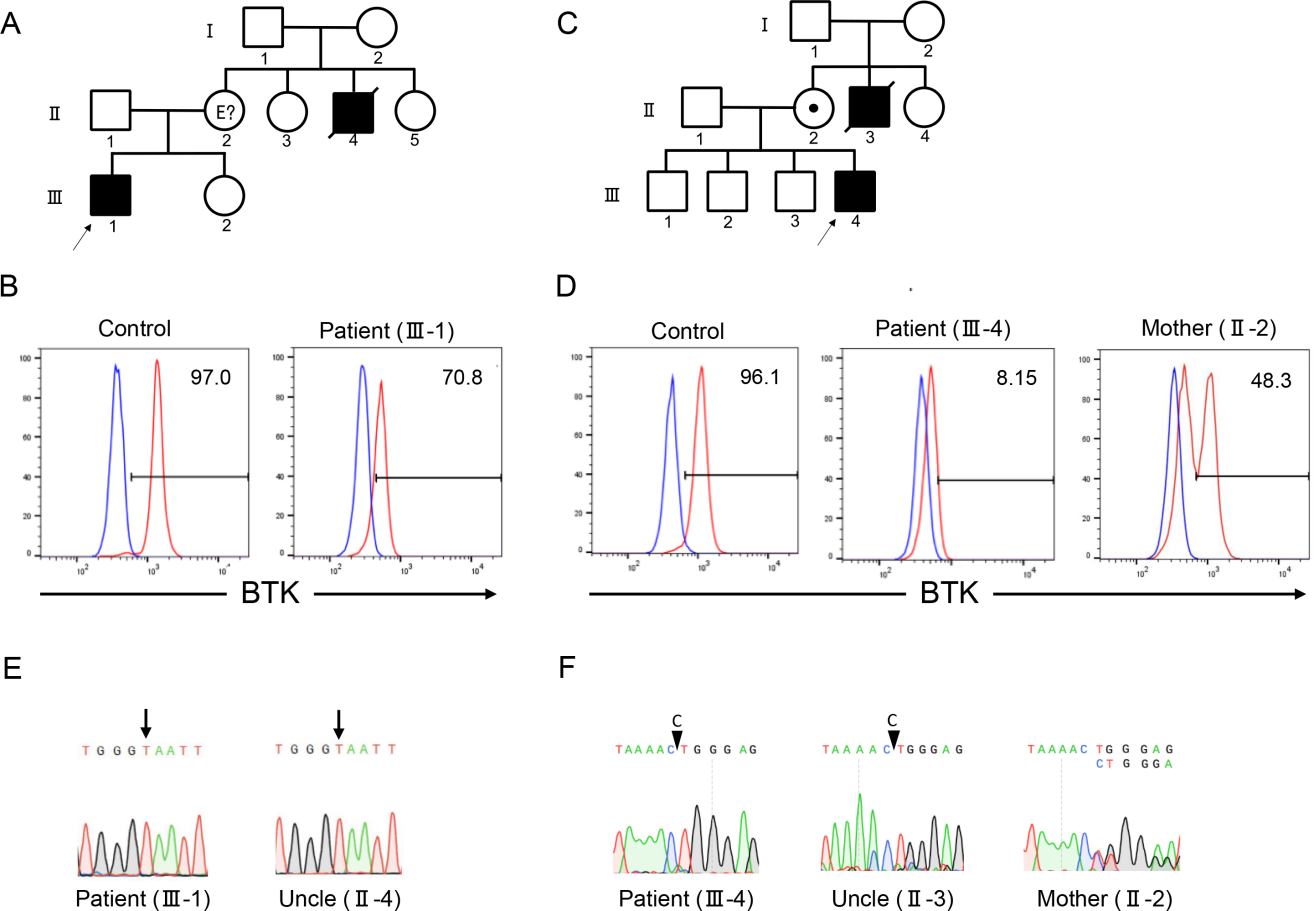
Family pedigrees, flow cytometry, and Sanger sequencing of patients 1 and 2. Family pedigrees of patients 1 **(A)** and 2 **(C)**. Flow cytometric analysis of Bruton tyrosine kinase (BTK) protein expression in monocytes revealed a decrease in expression levels in patients 1 **(B)** and 2 **(D)**. Red and blue lines indicate the staining of BTK monoclonal and isotype antibodies, respectively. Numbers indicate the percentages of the BTK-positive cells. Sanger sequencing revealed the presence of *BTK* variants c.1766A>T and c.530delC in patients 1 **(E)** and 2 **(F)**, respectively. The mother of patient 2 showed mosaic expression of the BTK protein **(D)** and *BTK* gene **(F)**.

Patient 1 (III-1) was a 6-year-old Japanese boy who presented with persistent fever lasting for a month. He was born to non-consanguineous parents, and his maternal uncle (II-4) died of sepsis at the age of three years (**Figure 1A**). The laboratory test results revealed hypogammaglobulinemia (IgG, 85 mg/dL; IgA, 18 mg/dL; and IgM, 12 mg/dL); specific antibodies against hepatitis B virus, rubella, and varicella were undetectable despite previous vaccination (**Supplementary Table 1**). Flow cytometric analysis of peripheral blood showed depletion of CD19^+^ cells (0.1% of total lymphocytes) (**Figure 1B**). The family history and B-cell-deficient antibody deficiency suggested that the patient had XLA. Flow cytometric analysis of BTK protein expression revealed a decrease in the BTK expression levels. Therefore, the patient was diagnosed with XLA.

Patient 2 (III-4) was a 2-year-old Japanese boy who presented with fever and skin eruptions. He was born to non-consanguineous parents and his maternal uncle (II-3) died of fever at the age of two years (**Figure 1C**). The serum immunoglobulin levels were low (IgG, 34 mg/dL; IgA, 10 mg/dL; IgM, 46 mg/dL); the titers of specific antibodies against measles, rubella, pertussis, and varicella were extremely low after vaccination (**Supplementary Table 1**). Lymphocyte subset analysis revealed depletion of CD19^+^ cells (0.08% of the total lymphocytes). The patient was suspected of having XLA because of the patient’s family history and B-cell deficiency. Flow cytometric analysis revealed a decrease in BTK protein expression levels in the patient. His mother (II-2) exhibited a bimodal pattern of BTK expression pattern, indicating that the mother was a carrier (**Figure 1D**).

### 3.2 Genetic findings

Patient 1 harbored c.1766A>T, p.Glu589Val variant of the *BTK* gene (**Figure 1E**). The deceased uncle harbored the same variant of *BTK* as that detected in the patient. Patient 2 harbored the c.530delC variant of the *BTK* gene (**Figure 1F**). Both variants were novel. The patient’s uncle harbored the same variant. Sanger sequencing of the patient’s mother revealed a double peak, indicating that the patient’s mother was a carrier.

## 4 Discussion

Although knowledge of family history is crucial for diagnosing an IEI, some family members may die during infancy or before a definite diagnosis is established. The diagnosis of an IEI as an underlying disease is important because infants are susceptible to a variety of infectious diseases, and in rare cases, these diseases may prove fatal for healthy children as well. In 21 countries (Australia, Argentina, Bangladesh, Brazil, China, Denmark, France, Germany, India, Indonesia, Japan, Korea, Malaysia, Mexico, Peru, Russia, Singapore, Tajikistan, Thailand, Uganda, USA), including Japan, preservation of the umbilical cord as a souvenir of birth is customary (3). Especially, in Japan, almost all families preserve the umbilical cord. Previously, preserved umbilical cords have been used to diagnose many congenital infectious diseases, such as cytomegalovirus, enterovirus, rubella, hepatitis B virus, herpes simplex virus, and parechovirus (3–8). Furthermore, monogenic defects have been diagnosed using umbilical cords; X-linked ectodermal dysplasia with immunodeficiency (NEMO deficiency) has also been recognized as an IEI (**Table 1**) (9–13). The findings of previous studies and our experience with the two patients indicate that dried umbilical cords can serve as diagnostic tools even after approximately 50 years (10, 13). In the future, we hope that genetic diagnosis using umbilical cords will be widely used to confirm accurate family history of inherited diseases for which family cases are ambiguous.

**Table 1:** nosis of monogenic diseases using preserved umbilical cords.

Disease	Age at death	Preservation	Diagnosis	Variant	Reference
SMA	infant	2 years	PCR targeting SMN exon 7,8 electrophoresis	*SMN* exon 7 telomeric deletion	9
DMD	14 years	38 years	Search for 4321 genes using NGS, Sanger sequence	*DMD* c.6292C>T	10
Pompe disease	11 months	2 years	Search for 4100 genes using NGS	*GAA* c.1939G>A	11
CDG	2 months	1 year	Search for 4100 genes using NGS	*RFT1* c.1209G>C	12
MAHCC	23 years	25 years	Whole-exome sequencing	*MMACHC* c.271dupA and c.347T>C	12
XL-EDA-ID	4 months	40 years	PCR targeting genetic variant in nephew, Sanger sequence	*IKBKG* c.262_264delGAG	13
	7 moths				

SMA, spinal muscular atrophy; DMD, Duchenne muscular dystrophy; CDG, congenital disorders of glycosylation; MAHCC, methylmalonic aciduria and homocystinuria, cobalamin C type; XL-EDA-ID, X-linked anhidrotic ectodermal dysplasia with immunodeficiency; NGS, next-generation sequencing.

The advantage of a confirmed diagnosis of a deceased individual is that the diagnosis helps determine not only family history and cause of death, but also prenatal and carrier diagnoses (9–11). If the first child dies before a definitive diagnosis is made, a postnatal genetic diagnosis is crucial: his or her diagnosis will influence the prenatal diagnosis of subsequent children. Whole-exome or whole-genome sequencing may be used for prenatal diagnosis; however, these are not routine tests and are usually performed only in cases of fetal abnormalities (15, 16). Thus, accurate genetic information is important for diagnosis of diseases such as IEIs that do not result in conspicuous structural abnormalities in the fetus. Regarding carrier diagnosis, targeted genetic testing is possible when accurate genetic information on affected individuals in the family is available. This reduces the risk of detection of unrelated genetic variants and provides detailed information, including details of the mosaic variants of target genes (10).

The quality of the DNA extracted from preserved umbilical cords should also be assessed. The extracted DNA may be degraded into small fragments of 200-3,000 bp (10) and may not be suitable for conducting long-distant PCR analysis (10, 13). Therefore, short-distant PCR and Sanger sequencing are chosen when the target variant is known, whereas short-read sequencing with next-generation sequencing is performed when the target variant is unknown. However, detection of large deletions and structural aberrations may not be possible using short-read sequencing. In our patients, the variants were a single nucleotide substitution and deletion, so the PCR products were short and could be amplified even though the PCR DNA was of poor quality. Fortunately, PCR products were obtained, and clear results were provided.

In conclusion, the deceased maternal uncles of two patients were diagnosed with IEIs based on the analysis of the deceased persons’ preserved umbilical cords. This study confirms the possibility of establishing a definitive genetic diagnosis of deceased individuals using their preserved umbilical cords.

## Data Availability

The datasets used in this study are not publicly available to protect participant/patient anonymity. Requests to access the datasets should be directed to the corresponding author.
