# Supplementary material for: Case Report: Preserved umbilical cords underscore family histories of inborn errors of immunity
Source: Front Immunol. 2025 Jul 8;16:1605857. doi: 10.3389/fimmu.2025.1605857 (PMC12279766; doi:10.3389/fimmu.2025.1605857)
Supplement: Supplementary file 1 [file Table1.docx]

**Supplementary Table 1. Laboratory studies**

|  | Patient 1 | Patient 2 | Unit | Normal range |
| --- | --- | --- | --- | --- |
| WBC | 17,700 | 10,400 | /μL | 5,000-14,500 |
| Neutrophils | 12,670 | 620 | /μL | 1,500-8,500 |
| Lymphocytes | 3,610 | 6,760 | /μL | 1500-7000 |
| Hematocrit | 39.2 | 35 | % | 34.8-43.0 |
| Hemoglobin | 13.2 | 11.9 | g/dL | 11.5-14.4 |
| Platelets | 55 | 62.9 | x 10^4^/μL | 18.0-51.0 |
| IgG | 85 | 34 | mg/dL | 667-1,179 |
| IgA | 18 | 10 | mg/dL | 66-120 |
| IgM | 12 | 46 | mg/dL | 79-169 |
| C3 | 138 | 43.3 | mg/dL | 65-135 |
| C4 | 30 | 134 | mg/dL | 13-35 |
| CH50 | 51 | 34.2 | U/mL | 30-45 |
| HBs Ab | 2.0 (-) | NE |  | + |
| Measles Ab | <2.0 (-) | 3.8 (±) |  | + |
| Rubella Ab | NE | 2.0 (-) |  | + |
| VZV Ab | <2.0 (-) | 3.1 (±) |  | + |
| Pertussis PT | NE | 3 | EU/mL | >10 |
| Pertussis FTA | NE | 23 | EU/mL | >10 |
| CD3 | 96.2 | 96.1 | % | 65.6-77.2 |
| CD4 | 43.1 | 65.9 | % | 31.7-54.7 |
| CD8 | 32.3 | 26.0 | % | 15.7-28.9 |
| CD19 | 0.1 | 0.082 | % | 5.8-11.7 |
| CD16^+^CD56^+^ | 2.4 | 2.88 | % | 2.4-10.2 |

WBC, white blood cells; Ig, immunoglobulin; HBs, hepatitis B surface; Ab, antibody; VZV, varicella-zoster virus; NE, not evaluated.

Values over and under the normal range are written in red and blue colors, respectively.


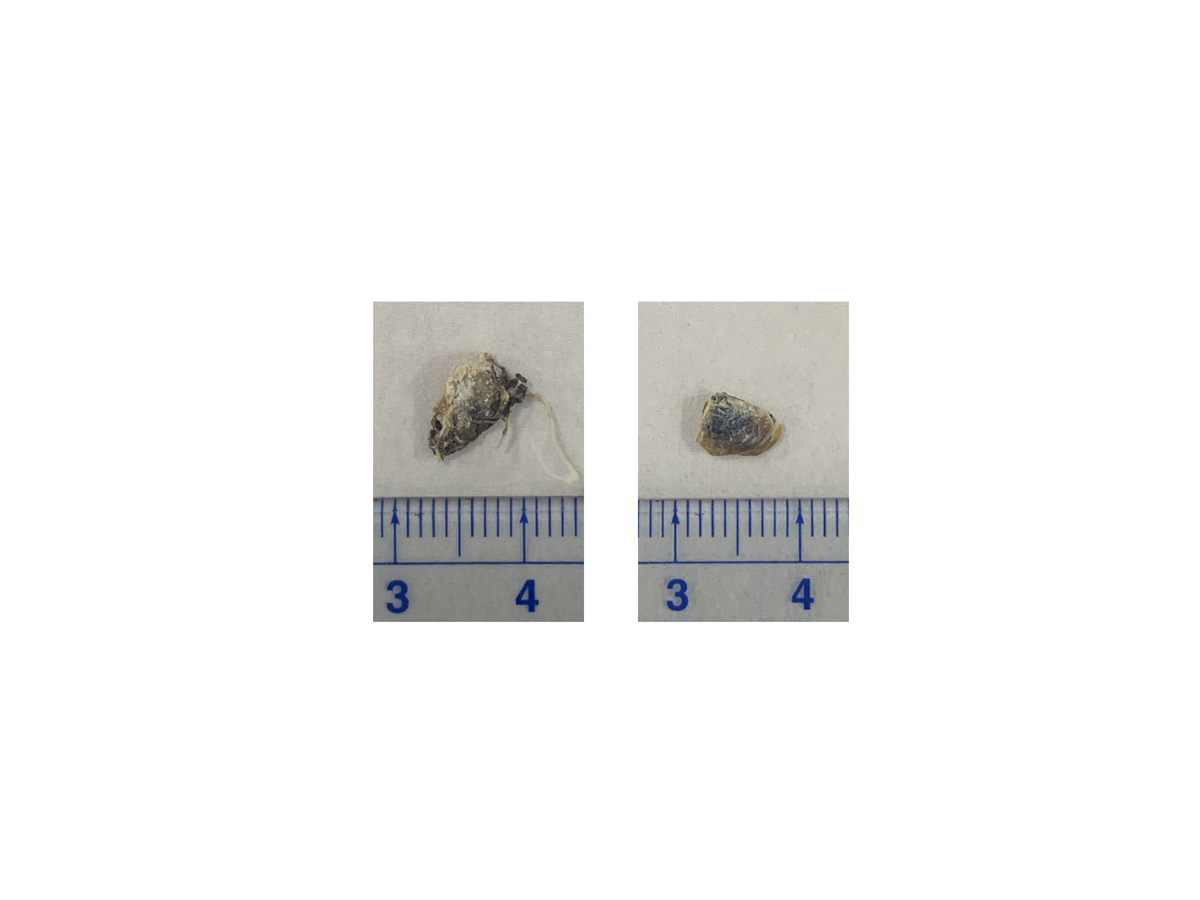


**Supplementary Figure 1. Preserved umbilical cord**

The left image shows the preserved umbilical cord of patient 1's maternal uncle (II-4) who died 48 years ago, while the right image shows that of patient 2’s maternal uncle (II-3) who died 49 years ago.
